# HeartSmart: development and evaluation of a culturally tailored, theory-based cardiovascular health promotion programme for university students in a North-Central Nigerian university — a quasi-experimental pre-post study

**DOI:** 10.1186/s12889-026-27944-7

**Published:** 2026-05-28

**Authors:** Olasunkanmi Samson Coker, Edward A. Omudu, Adamu Ishaku Akyala, Chukwu O.O. Chukwu, Regina Bolutife Coker, Awayimbo R. Jaggu, Salako Ezekiel Oluseye, John Etim Edet

**Affiliations:** 1 Global Health and Infectious Disease Control Institute, Nasarawa State University, Keffi, Nasarawa State Nigeria; 2 Department of Epidemiology and Disease Control Technology, Federal College of Veterinary and Medical Laboratory Technology (FCVMLT), Vom, Plateau State Nigeria; 3 Department of Biological Sciences, Benue State University, Makurdi, Benue State Nigeria; 4 Department of Health Education, Smart Gems Academy, Kuru, Nigeria

**Keywords:** Cardiovascular disease, Health promotion, Cultural tailoring, University students, Nigeria

## Abstract

**Background:**

Cardiovascular disease (CVD) is the leading cause of mortality in sub-Saharan Africa, with hypertension as the primary modifiable risk factor. Despite a high and largely undetected hypertension burden, no culturally tailored, theory-based cardiovascular health promotion programme has been developed or evaluated for university students in North-Central Nigeria. This study reports the development and evaluation of the HeartSmart Programme, designed to address documented deficits in CVD knowledge, risk perception, and behavioural intentions.

**Methods:**

A quasi-experimental single-group pre-test/post-test design was employed. Fifty purposively selected high-risk undergraduate students at the University of Jos (UNIJOS) completed validated ABCD Risk Perception Questionnaire assessments on the programme day. The HeartSmart Programme — a one-day, peer-delivered intervention comprising six evidence-based modules — was developed through systematic needs assessment grounded in Phase 1 baseline findings (*N* = 1,300) and clinical cardiovascular assessment. Three theoretical frameworks (Health Belief Model, Social Cognitive Theory, Theory of Planned Behaviour) were explicitly operationalised into programme content. Paired-samples t-tests and Cohen’s d quantified pre-post changes.

**Results:**

Significant improvements were observed across all four ABCD domains (all p ≤ 0.013). CVD knowledge demonstrated a large effect (d = 1.42; 73.0% to 93.3%; p < 0.001), with ‘good knowledge’ increasing from 26.0% to 90.0% of participants. Perceived benefits/exercise intentions showed a medium-large effect (d = 0.73; *p* < 0.001). Healthy eating intentions showed a small-to-medium effect (d = 0.48; *p* = 0.001). Perceived risk showed a small but significant effect (d = 0.36; *p* = 0.013). Programme fidelity was excellent: 98% content coverage, 100% retention, and satisfaction 4.74/5.00.

**Conclusions:**

HeartSmart demonstrates that a brief, theory-based, culturally tailored CVD health promotion programme can achieve large knowledge improvements and meaningful attitudinal changes in high-risk university students at a North-Central Nigerian university. Findings are limited to immediate post-programme effects; longitudinal research is needed to assess behavioural and clinical translation. The programme offers a replicable, low-resource model for primordial CVD prevention in sub-Saharan African university settings.

**Supplementary Information:**

The online version contains supplementary material available at 10.1186/s12889-026-27944-7.

## Background

### Burden of CVD in the Nigerian context

Cardiovascular disease (CVD) is responsible for approximately 17.9 million deaths annually, representing 32% of all global mortality, with more than 80% of these deaths occurring in low- and middle-income countries (LMICs) [1]. In sub-Saharan Africa, the epidemiological transition toward non-communicable diseases — driven by urbanisation, dietary change, and physical inactivity — has placed CVD at the centre of public health planning [2]. Nigeria, as Africa’s most populous LMIC, exemplifies this transition acutely: CVD accounts for approximately 11% of all national mortality, and hypertension — the single most important modifiable cardiovascular risk factor — has reached epidemic proportions, with national prevalence estimates of 30–33% among adults [3, 4]. Critically, fewer than 40% of those affected are aware of their condition, fewer than 20% receive treatment, and fewer than 5% achieve adequate blood pressure control [3], reflecting a healthcare system with severely limited capacity for systematic cardiovascular screening and prevention.

### University student vulnerability

University students represent a population of particular strategic importance for primordial CVD prevention. Behaviours and risk perceptions established during the university years track powerfully into middle age [5], and prospective cohort data confirm that childhood and adolescent cardiovascular risk factors — elevated blood pressure, dyslipidaemia, smoking — predict subclinical and clinical cardiovascular events in adulthood after follow-up periods exceeding 30 years [6]. Nigerian university students face a distinctive constellation of cardiovascular risk exposures: transition from structured family meals to irregular dietary patterns determined by campus food vendor availability; sedentary academic routines replacing habitual physical activity; chronic psychosocial stress from academic pressure and financial insecurity; and near-complete absence of systematic cardiovascular health monitoring within university health services [7, 8].

Clinical evidence confirms a substantial and largely undetected hypertension burden in this population. A clinical assessment conducted as part of the present research found that 26% of purposively selected high-risk University of Jos students met WHO 2021 hypertension criteria (≥ 140/90 mmHg), rising to 50% under ACC/AHA 2017 criteria (≥ 130/80 mmHg), with complete unawareness in all cases — a 100% within-sample detection gap under both classification systems [9]. Comparable hypertension burden has been documented among university students in other Nigerian geopolitical zones, with pre-hypertension and hypertension rates of 22–50% reported in south-east Nigerian universities [10]. A further dimension specific to sub-Saharan African populations is the well-documented pattern of salt-sensitive hypertension, operating independently of excess body weight through exaggerated renal sodium retention and heightened vascular reactivity [11, 12]. This was confirmed in the clinical assessment: 76% of participants meeting hypertension criteria had normal BMI [9], establishing that obesity-centred cardiovascular risk frameworks are insufficient for this population.

### Evidence gaps and intervention readiness

The broader cognitive landscape among Nigerian university students compounds this clinical challenge. A companion cross-sectional survey (*N* = 1,300) documented that CVD knowledge, while moderate (62% correct response rate), harboured critical gaps: only 26.8% of students correctly distinguished HDL from LDL cholesterol, and only 35.3% recognised family history as a major CVD risk factor [13]. More consequentially, CVD knowledge and perceived personal CVD risk were functionally uncorrelated (*r* = − 0.029, *p* = 0.313), confirming a knowledge–risk perception disconnect that challenges the information-deficit model underlying traditional health education programmes [13, 14]. Students expressed moderately high intentions for exercise and healthy eating, suggesting motivational readiness, yet lacked the skills, confidence, and practical knowledge to convert these intentions into concrete action — the classic intention–behaviour gap.

Despite these documented needs, a critical intervention gap persists. The few educational interventions attempted in Nigerian university settings have been overwhelmingly atheoretical, culturally generic, and evaluated only against knowledge outcomes — neglecting the psychological constructs of perceived risk and behavioural intention that are more proximally predictive of health behaviour change [15]. No published, culturally tailored, theory-driven cardiovascular health promotion programme designed specifically for North-Central Nigerian university students exists in the peer-reviewed literature. This setting — characterised by near-universal religious affiliation, ethnically diverse student populations at state and federal universities, and the practical constraints of hostel living and limited student budgets — requires deep-structure cultural adaptation that transplanted Western or generic programmes cannot provide [16, 17].

Three characteristics establish this population as intervention-ready: first, moderate existing knowledge provides a foundation for targeted gap-filling; second, moderately high stated behavioural intentions indicate motivational readiness, with the primary barrier being practical rather than volitional; and third, near-universal religious affiliation provides a culturally resonant normative framework through which health behaviour change can be authentically motivated. A one-day, peer-delivered intensive format was selected on the basis of: (a) demonstrated effectiveness in comparable resource-limited university settings [18]; (b) the concentration of motivated participation on a single occasion, overcoming the attendance attrition that undermines multi-session programmes in Nigerian university contexts; and (c) feasibility within the resource constraints of Nigerian university health services, enabling scale-up without specialist external staffing.

The HeartSmart Programme was developed to address this gap comprehensively, grounded in a three-phase sequential explanatory mixed-methods design. This paper reports the development rationale and pre-post effectiveness evaluation of the HeartSmart Programme.

## Methods

### Study design and setting

A quasi-experimental single-group pre-test/post-test design was employed to evaluate the HeartSmart Programme. This design was selected for two reasons: first, the purposively targeted high-risk sample — all of whom were unaware of their hypertension status — rendered withholding the intervention ethically unjustifiable under the Declaration of Helsinki; second, this design is consistent with established practice for proof-of-concept evaluations of novel, culturally tailored health promotion programmes in resource-limited settings [18, 19]. The absence of a concurrent control group is acknowledged as a limitation (see Limitations section).

The study was conducted at the University of Jos (UNIJOS), Jos, Plateau State, North-Central Nigeria — a federal university with an undergraduate enrolment of approximately 40,000 students. UNIJOS was selected as the intervention site for the following reasons: (a) Phase 1 baseline survey and clinical assessment data were available from UNIJOS participants, as both the Phase 1 cross-sectional survey and the clinical assessment were conducted prospectively by the same research team as part of this sequential mixed-methods programme of research — not from pre-existing secondary records — enabling needs-based programme development grounded in population-specific primary evidence [9, 13]; (b) the clinical detection findings — 26% hypertension prevalence with a 100% detection gap [9] — established the most urgent unmet need; and (c) logistical access to the participant pool identified through Phase 1 screening was established. Pre-test and post-test assessments were conducted on the same programme day, bracketing delivery, to minimise confounding from intervening experiences and to ensure 100% paired data collection. The companion Nasarawa State University, Keffi (NSUK) site was reserved for replication in future research. Data collection took place during the 2024/2025 academic session.

### Participants, eligibility, and recruitment

Eligibility criteria for participation were: (a) full-time undergraduate enrolment at UNIJOS; (b) age 18–35 years (a precautionary upper bound reflecting that the actual participant age range was 20–27 years, consistent with standard undergraduate enrolment at Nigerian federal universities); (c) presence of at least one established cardiovascular risk indicator — family history of CVD in a first-degree relative, overweight/obese BMI (≥ 25 kg/m²), or self-reported physical activity below WHO-recommended levels (< 150 min/week) — with any single indicator sufficient for eligibility; and (d) ability to provide informed consent. Postgraduate students, part-time students, and those with a pre-existing diagnosed cardiovascular condition were excluded.

**Fig. 1:**
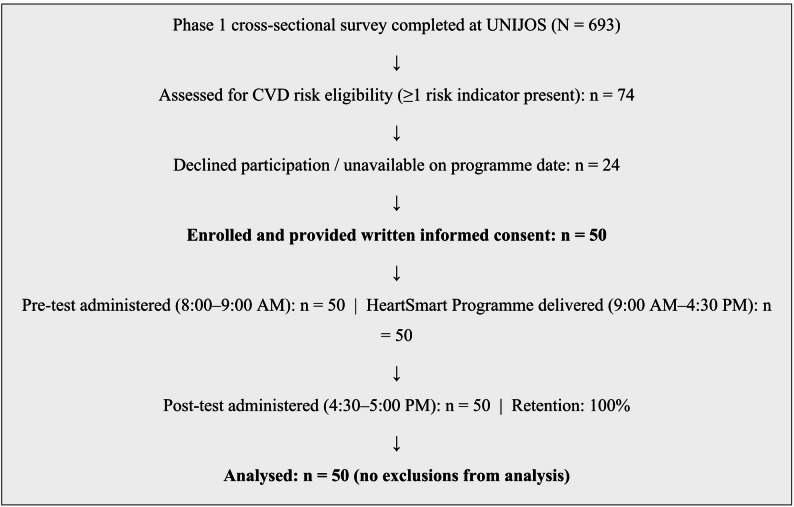
TREND flow diagram of HeartSmart Programme activities

Participants were purposively recruited from the pool of 693 UNIJOS students who completed the Phase 1 cross-sectional survey [13]. From this pool, 74 students were identified as meeting the cardiovascular risk eligibility criteria (at least one of the three risk indicators above). All 74 were formally invited to participate in the HeartSmart Programme. Of these, 24 declined participation or were unavailable on the programme date, yielding 50 participants who provided written informed consent and completed the full programme day. This recruitment process is illustrated in Fig. 1 (TREND flow diagram). There was no attrition on the programme day: all 50 participants completed both pre-test and post-test assessments, yielding 100% data completeness for the primary analyses.

Purposive sampling was the appropriate strategy for this quasi-experimental intervention study, in which the primary objective was to evaluate programme effectiveness in those most in need. Sample size determination was criterion-based rather than formula-driven: *n* = 50 represents the complete eligible, consenting pool meeting all inclusion criteria from the Phase 1 sample. This approach is consistent with published quasi-experimental health promotion studies in similar settings, where purposive samples of 30–60 high-risk individuals are standard for initial proof-of-concept evaluation [18, 19].

### TREND flow diagram

Figure 1 presents the participant flow following Transparent Reporting of Evaluations with Nonrandomized Designs (TREND) guidelines.

### Baseline clinical assessment

Prior to the programme day, all 50 participants underwent clinical cardiovascular assessment over three preceding days. Blood pressure was measured using validated automated sphygmomanometers (Omron HEM-7120 or equivalent validated upper-arm devices) by trained nursing staff following standardised WHO/ISH protocols: five-minute seated rest before measurement; two readings at 1–2 min intervals; a third reading taken if the first two differed by > 10 mmHg, with the mean of the two closest values recorded. Blood pressure was classified using WHO 2021 guidelines as the primary standard and ACC/AHA 2017 guidelines as secondary comparison [9]. Body mass index (kg/m²) was calculated from height and weight. Fasting capillary blood glucose was measured after an 8–10 h fast. All participants received individual clinical results communicated via the HeartSmart Traffic Light Risk System. Written referral letters were issued to all participants meeting WHO 2021 hypertension criteria (BP ≥ 140/90 mmHg).

### Needs assessment and programme design rationale

The HeartSmart Programme’s content priorities were driven entirely by Phase 1 baseline findings rather than generic cardiovascular curricula. Three key findings shaped programme architecture: (1) the 100% within-sample detection gap established personalised clinical feedback as the most critical risk-perception activation mechanism; (2) the identification of HDL/LDL cholesterol literacy (only 26.8% correct) and family history recognition (only 35.3% correct) as the most prevalent knowledge gaps directed specific module content; and (3) the high prevalence of sedentary behaviour (> 60% below WHO guidelines) contrasted with moderate-to-high stated behavioural intentions, indicated that the primary barrier to behaviour change was practical rather than motivational [13].

### Theoretical framework

Programme development was grounded in an integrated framework incorporating three complementary health behaviour theories. The Health Belief Model (HBM) [20] provided the framework for addressing perceived susceptibility, perceived severity, perceived benefits, perceived barriers, and cues to action. The Social Cognitive Theory (SCT) [21] informed strategies to develop self-efficacy through mastery experience, vicarious learning, and social modelling. The Theory of Planned Behaviour (TPB) [22] framed the programme’s primary outcome constructs and provided the theoretical basis for targeting subjective norms through faith-based and collectivist cultural frameworks. Table 1 presents the explicit mapping of theoretical constructs to intervention components and outcome measures.

**Table 1:** Theory–construct–intervention component–outcome mapping

Theory	Key Construct	HeartSmart Component	Outcome Measured
Health Belief Model	Perceived Susceptibility	Traffic Light Risk System; personalised BP feedback (Module 1)	Perceived CVD Risk (ABCD-B)
	Perceived Severity	CVD epidemiology in Nigeria; complication narratives (Module 1)	CVD Knowledge (ABCD-A)
	Perceived Benefits	Benefits of diet, exercise, stress reduction (Modules 2–4)	Perceived Benefits/Exercise Intentions (ABCD-C)
	Cues to Action	Clinical feedback; faith-based stewardship; peer educator delivery (Module 1)	CVD Knowledge; Perceived Risk (ABCD-A, B)
Social Cognitive Theory	Self-Efficacy	Campus-specific barrier problem-solving; hostel-adaptable exercise (Modules 3, 6)	Perceived Benefits/Exercise Intentions (ABCD-C)
	Observational Learning	Peer educator delivery; group demonstrations (Modules 2–4)	CVD Knowledge; Healthy Eating Intentions (ABCD-A, D)
	Self-Regulation	SMART goal worksheets; lapse management strategies (Module 6)	Healthy Eating Intentions; Exercise Intentions (ABCD-C, D)
Theory of Planned Behaviour	Attitudes	Benefits framing; outcome expectancy demonstrations (Modules 2–4)	Perceived Benefits/Exercise Intentions (ABCD-C)
	Subjective Norms	Islamic amanah and Christian stewardship frameworks; collectivist family framing (Modules 1, 5)	Perceived CVD Risk; Perceived Benefits (ABCD-B, C)
	Behavioural Intention / Implementation Intentions	IF-THEN planning; social accountability partner commitment ceremony (Module 6)	Healthy Eating Intentions; Exercise Intentions (ABCD-C, D)

*ABCD* ABCD Risk Perception Questionnaire subscales (A = CVD Knowledge; B=Perceived CVD Risk; C=Perceived Benefits/Exercise Intentions; D=Healthy Eating Intentions). *HBM* Health Belief Model, *SCT* Social Cognitive Theory, *TPB* Theory of Planned Behaviour

### Programme description

The HeartSmart Programme was delivered as a single intensive day-long workshop (8:00 AM to 5:00 PM, approximately seven hours of instructional content with structured breaks). The one-day intensive format was deliberately chosen for three reasons: (a) multi-session programme designs are associated with high attrition in Nigerian university contexts due to academic schedule conflicts and transport barriers; (b) a single concentrated session maximises participant engagement intensity and enables peer-group dynamics to build progressively across modules; and (c) the format is deliverable within the resource envelope of university health services without specialist external staffing. This design is consistent with single-session intensive health promotion models that have demonstrated significant knowledge and attitude improvements in comparable resource-limited settings [18, 19]. The programme comprised six integrated modules, described below and summarised in Table 2.

**Table 2:** HeartSmart Programme module structure, theoretical alignment, and baseline findings addressed

Module (Duration)	Time	Core Content	Theoretical Basis	Baseline Finding Addressed
1: CVD Knowledge & Risk Factors	60 min	CVD epidemiology in Nigeria; HDL/LDL cholesterol; family history; hypertension classification (WHO 2021 primary; ACC/AHA 2017 secondary); personalised clinical feedback (Traffic Light); faith-based stewardship framing	HBM: Perceived Susceptibility & Severity; Cues to Action	62% knowledge rate; only 26.8% knew HDL/LDL; 35.3% recognised family history; 100% unaware of hypertension status
2: Nutrition for Heart Health	90 min	Dietary sodium & salt-sensitive hypertension; Nigerian Heart-Healthy Plate; budget-conscious local food substitutions; saturated fat and sugar reduction	TPB: Attitude & Behavioural Intention; SCT: Self-Efficacy (dietary domain)	High intentions without practical skills; BMI–BP paradox; salt-sensitive hypertension pathway
3: Physical Activity & Exercise	60 min	WHO 150 min/week guideline; campus-specific, hostel-compatible exercises; barrier identification and peer problem-solving	SCT: Self-Efficacy (mastery & vicarious learning); TPB: Perceived Behavioural Control	> 60% sedentary; moderate-high exercise intentions; practical barrier gap
4: Managing Stress & Mental Health	60 min	HPA axis and BP mechanisms; deep breathing; progressive muscle relaxation; time management; campus counselling referral	HBM: Perceived Severity; SCT: Behavioural Self-Regulation	Academic stress as cardiovascular risk driver; low perceived risk despite clinical evidence
5: Tobacco, Alcohol & Substance Use	45 min	CVD consequences of tobacco/alcohol; gender asymmetry (male OR = 10.17 smoking); faith/social norm leveraging; resistance strategies	TPB: Subjective Norms; HBM: Cues to Action	Religious norms protective; strong gender disparity in substance use risk
6: Action Planning & Sustaining Change	45 min	SMART goal-setting worksheets; implementation intentions (IF-THEN planning); lapse management; social accountability partner; group commitment ceremony	SCT: Self-Regulation & Behavioural Self-Efficacy; TPB: Intention Formation	High intentions without concrete plans; knowledge-to-behaviour gap

Traffic Light thresholds: Red = BP ≥ 140/90 mmHg (referral); Amber = BP 120–139/80–89 mmHg (lifestyle modification); Green = BP < 120/<80 mmHg (positive reinforcement)

*HBM* Health Belief Model, *SCT* Social Cognitive Theory, *TPB* Theory of Planned Behaviour

### Cultural tailoring

Cultural tailoring was conducted at both surface and deep structural levels [16]. Surface structure tailoring incorporated: culturally familiar examples and locally relevant Nigerian foods (moi-moi, beans, ogi, garden egg, green leafy vegetables); photographs and scenarios from the UNIJOS campus environment; and standard English supplemented with North-Central Nigerian idiomatic expressions. Deep structure tailoring addressed cultural values, social norms, and faith-based normative frameworks. With 98% of Phase 1 survey participants identifying with either Islam or Christianity, Islamic amanah (the body as a divine trust) and Christian understandings of the body as a temple of the Holy Spirit were integrated into Module 1. Economic tailoring ensured all dietary recommendations were benchmarked against typical student food budgets at UNIJOS hostel canteens.

### Programme delivery

The programme was delivered by two final-year health sciences students from UNIJOS who had undergone comprehensive two-day training, supplemented by the principal investigator and two trained research assistants. The peer educator model was adopted on the basis of consistent evidence that peer-delivered health promotion produces superior knowledge, attitude, and intention outcomes in university settings [23]. Delivery employed interactive presentations, facilitated group discussions, small-group problem-solving activities, practical demonstrations, and individual goal-setting exercises. Printed educational materials were distributed to all participants.

### Outcome measures

The primary outcome instrument was the ABCD Risk Perception Questionnaire [14], a validated tool assessing four cardiovascular health constructs: (A) CVD Knowledge (8 binary items; score 0–8; percentage correct reported); (B) Perceived CVD Risk (items 9–16; scale 8–32); (C) Perceived Benefits/Exercise Intentions (items 17–23; scale 7–28); and (D) Healthy Eating Intentions (items 24–26; scale 3–12). Subscales C and D operationalise behavioural intention — the proximal predictor of behaviour in the TPB framework — through Likert-rated statements about intentions to exercise and eat healthily, respectively. The instrument was used without modification to dietary item content: following expert panel review by a nutritionist, public health physician, and health promotion specialist, the panel confirmed that the original ABCD items were appropriate for the Nigerian university context without requiring textual changes. The full questionnaire instrument, including all 26 ABCD items and the demographic and health behaviour items comprising the complete study questionnaire, is provided as Appendix A.

### Evaluation procedure

On the programme day, all 50 participants completed the ABCD Questionnaire as a pre-test (8:00–9:00 AM) before any programme content was delivered, under standardised conditions with research assistants available for item clarification. The HeartSmart Programme was delivered from 9:00 AM to approximately 4:30 PM. The post-test — identical to the pre-test — was administered immediately after programme completion (4:30–5:00 PM). To minimise social desirability bias, the post-test was administered by research assistants who had not been involved in programme content delivery. In addition, the pre-test was administered before any programme content was delivered, and participants were not informed that a post-test would follow, reducing priming for specific questionnaire items. The same-day pre-test/post-test design was deliberately employed to: (a) maximise paired data completeness; (b) capture the full immediate educational impact of the intervention; and (c) ensure both assessments occurred under comparable environmental conditions. The limitations of this design — including the risk of testing effects and the Hawthorne effect — are fully acknowledged in the Limitations section.

### Statistical analysis

The primary analysis compared pre- and post-programme ABCD subscale scores using paired-samples t-tests, with significance set at α = 0.05. Effect sizes were calculated as Cohen’s d, interpreted as small (d = 0.20–0.49), medium (d = 0.50–0.79), or large (d ≥ 0.80) [24]. McNemar’s test compared pre- and post-programme categorical outcomes. Normality was assessed using the Shapiro-Wilk test and visual inspection of histograms and Q-Q plots; Wilcoxon signed-rank tests were used for non-normally distributed differences. All analyses were conducted using IBM SPSS Statistics Version 29.0.

### Ethical considerations

Ethical approval was obtained from the Plateau State Ministry of Health Research Ethics Committee prior to data collection. Institutional permissions were obtained from UNIJOS and NSUK. All activities complied with the Declaration of Helsinki (2013) [25] and Nigerian National Health Research Ethics Committee guidelines. All participants provided voluntary written informed consent prior to any study activity. Participants with elevated or hypertensive blood pressure results received written referral letters to the UNIJOS Health Centre with follow-up instructions.

## Results

### Participant characteristics

The 50 UNIJOS intervention participants had a mean age of 23.12 ± 1.79 years (minimum 20, maximum 27 years). The sample was predominantly male (64.0%, *n* = 32). Faculties represented included Natural Sciences (40.0%), Medical Sciences (28.0%), Engineering (16.0%), and other faculties (16.0%). Religious affiliations were Christianity (68.0%) and Islam (32.0%). All participants were unmarried. Risk indicators at enrolment: family history of CVD (60.0%), overweight/obese BMI (24.0%), and sedentary lifestyle (78.0%), with multiple risk indicators present in 52.0% of participants.

### Baseline clinical findings

Baseline clinical assessment revealed a clinically significant cardiovascular risk profile. Mean systolic blood pressure was 129.2 ± 12.76 mmHg and mean diastolic 82.1 ± 9.48 mmHg. Using WHO 2021 primary criteria, 26.0% (*n* = 13) met hypertension criteria (≥ 140/90 mmHg), with an additional 22.0% in the Normal-High category and 24.0% Elevated. Using ACC/AHA 2017 secondary criteria, 50.0% (*n* = 25) met hypertension thresholds. All participants meeting hypertension criteria were entirely unaware of their condition, representing a 100% within-sample detection gap. Mean BMI was 23.55 ± 4.59 kg/m²; 24.0% were overweight or obese; 76.0% of hypertensive participants had normal BMI. Fasting blood glucose was normal in 98.0% of participants (mean 4.62 ± 0.31 mmol/L). Full baseline clinical data are reported in the companion clinical assessment paper [9].

### Programme fidelity

Implementation fidelity was assessed by the principal investigator against a pre-specified fidelity checklist: 98% content coverage was achieved (one minor element of Module 5 abridged due to time constraints); 100% of planned activities were completed; 100% cultural sensitivity adherence was maintained; and 100% participant retention was achieved. Mean overall participant satisfaction was 4.74/5.00 (SD = 0.39), and 100% of participants indicated they would recommend the programme to peers.

### Intervention effectiveness

Table 3 presents the pre- and post-programme ABCD subscale comparisons. The HeartSmart Programme produced statistically significant improvements on all four outcome domains (all *p* ≤ 0.013).

**Table 3:** Pre- and Post-Intervention Comparison Across ABCD Outcome Domains (*n* = 50)

Domain (Scale)	Pre-test M (SD)%	Post-test M (SD)%	Mean Δ	t (49)	*p*	d	Effect Size	Decision
CVD Knowledge (ABCD-A; 0–8)	73.00 (11.67)	93.25 (10.17)	+ 20.25 pp	10.04	< 0.001	**1.42**	Large	H₀ Rejected
Perceived CVD Risk (ABCD-B; 8–32)	37.94 (7.76)	43.62 (15.16)	+ 5.69 pp	2.57	0.013	0.36	Small	H₀ Rejected
Perceived Benefits/Exercise Intentions (ABCD-C; 7–28)	78.79 (13.58)	86.43 (8.84)	+ 7.64 pp	5.14	< 0.001	0.73	Medium-Large	H₀ Rejected
Healthy Eating Intentions (ABCD-D; 3–12)	69.17 (12.74)	76.50 (12.79)	+ 7.33 pp	3.39	0.001	0.48	Small-Medium	H₀ Rejected

Effect sizes per Cohen (1988) [24]: Small d = 0.20–0.49; Medium d = 0.50–0.79; Large d ≥ 0.80. All improvements statistically significant (all *p* ≤ 0.013). 95% CI for knowledge change: [16.20, 24.30]. Good knowledge (76–100% correct) increased from 26.0% to 90.0% post-intervention (McNemar’s test, *p* < 0.001). ABCD-C and ABCD-D operationalise behavioural intention per the TPB framework

*pp* percentage points

Bold value indicates the largest effect size observed across all outcome domains

CVD Knowledge demonstrated the largest effect (d = 1.42, large), with mean scores increasing from 73.0% to 93.3%, a mean gain of 20.25% points (95% CI: 16.20–24.30; t(49) = 10.04, p < 0.001). The proportion in the ‘good knowledge’ category (76–100% correct) increased from 26.0% to 90.0% (McNemar’s test, *p* < 0.001).

Perceived Benefits/Exercise Intentions demonstrated a medium-large effect (d = 0.73), with mean scores increasing from 78.8% to 86.4%, a mean gain of 7.64% points (t(49) = 5.14, *p* < 0.001).

Healthy Eating Intentions demonstrated a small-to-medium effect (d = 0.48), with mean scores increasing from 69.2% to 76.5%, a mean gain of 7.33% points (t(49) = 3.39, *p* = 0.001).

Perceived CVD Risk demonstrated a small but statistically significant effect (d = 0.36), with mean scores increasing from 37.9 to 43.6 on the 8–32 scale (mean gain = 5.69 pp; t(49) = 2.57, *p* = 0.013). The perceived risk data revealed a clinically meaningful polarisation pattern post-intervention: the proportion with high perceived risk increased from 2.0% to 14.0%, while the proportion with low perceived risk also increased from 6.0% to 16.0%, both at the expense of the moderate-risk category.

The hierarchy of effect sizes — CVD knowledge (d = 1.42) > perceived benefits (d = 0.73) > healthy eating intentions (d = 0.48) > perceived risk (d = 0.36) — is theoretically coherent: cognitive outcomes are most readily modified in a single educational encounter, followed by attitudinal and motivational-volitional outcomes, with risk perception requiring personalised experiential input to shift.

## Discussion

### Summary of principal findings

The HeartSmart Programme is, to the authors’ knowledge, the first published systematically evaluated, culturally tailored cardiovascular health promotion programme designed specifically for and delivered to university students at a North-Central Nigerian university. The programme achieved statistically significant improvements across all four cardiovascular health outcome domains assessed by the ABCD Questionnaire, with the CVD knowledge effect size (d = 1.42) substantially exceeding benchmarks from systematic reviews of comparable interventions [26, 27, 32], and with meaningful attitudinal and motivational changes extending beyond factual knowledge. Implementation fidelity was excellent across all monitored indicators, and participant retention was complete.

### CVD knowledge: effect size in context

The large knowledge effect (d = 1.42) reflects the synergistic impact of theory-driven design, cultural tailoring, and explicit targeting of identified knowledge gaps. Content was derived from measured population-specific knowledge gaps — the HDL/LDL and family history items were given targeted attention based on baseline findings. Personalised clinical feedback activated the HBM’s cues to action construct through direct personal relevance. The peer educator model — delivering 98% fidelity — amplifies knowledge acquisition through heightened source credibility and reduced power differentials [23]. The faith-based framing provided a culturally resonant rationale for attending to cardiovascular health information that generic programmes lack.

This effect size exceeds the 0.30–0.60 range reported in systematic reviews of cardiovascular health education in young adult populations [26, 27] and is consistent with the principle that interventions targeting specific, identified knowledge deficits outperform generic CVD education. Among comparable studies in Nigerian university contexts, Odunaiya et al. [28] reported significant but smaller knowledge gains (d ≈ 0.45) from a multi-session programme, attributing the lower effect to generic rather than needs-assessment-derived content — supporting the HeartSmart approach. The present study advances prior Nigerian and African university-based CVD intervention research [28, 29] in three distinct ways: theoretical sophistication (explicit operationalisation of HBM, SCT, and TPB rather than atheoretical delivery); deep-structure cultural tailoring (faith-based frameworks and campus-specific practical guidance); and outcome breadth (measuring knowledge, risk perception, and two behavioural intention domains simultaneously).

### Behavioural intention: assessment and clinical significance

Behavioural intention was assessed through ABCD Questionnaire subscales C (Perceived Benefits/Exercise Intentions; 7 Likert-rated items, scale 7–28) and D (Healthy Eating Intentions; 3 Likert-rated items, scale 3–12). These subscales comprise statements rated on a 1–4 Likert scale about the participant’s intention to engage in specific physical activity and dietary behaviours, consistent with the TPB framework [22]. The medium-large effect on perceived benefits/exercise intentions (d = 0.73) confirms attitudinal and motivational change beyond factual knowledge acquisition. This is clinically important because behavioural intention — not knowledge — is the proximal predictor of behaviour change in the TPB framework, and because perceived benefits and self-efficacy are the constructs whose enhancement most reliably predicts subsequent health behaviour in the HBM and SCT [20, 21, 30].

### Knowledge–risk perception disconnect: programme implications

The companion survey study established a near-zero correlation between CVD knowledge and perceived personal risk (*r* = − 0.029, *p* = 0.313) across 1,300 students [13]. Dual-process theories of cognition offer the most parsimonious explanation: factual CVD knowledge is encoded through deliberative analytical processing (System 2), while personal risk perception is driven by affective experiential processing (System 1), responding to vivid personal experiences rather than abstract statistical information [31]. The present results demonstrate that personalised clinical feedback — delivered through the Traffic Light Risk System in the context of HBM engagement — provides the experiential input necessary to engage System 1 risk appraisal, achieving a small but significant risk perception effect where prior studies relying on information provision alone have produced null results [14, 15].

### Cultural tailoring: mechanisms of effect

Deep-structure cultural tailoring consistently produces superior engagement and behaviour change relative to standard interventions [16, 17]. The faith-based health stewardship framework is the most distinctive and likely most influential tailoring element for this population: by grounding cardiovascular self-care in Islamic amanah and Christian theology — value systems shared by 98% of participants — the programme converted abstract biomedical recommendations into obligations aligned with participants’ deepest normative commitments, engaging TPB’s subjective norms construct authentically. The dietary and physical activity tailoring directly addressed the practical barrier between intentions and behaviour identified at baseline, consistent with SCT’s self-efficacy mechanism.

### Limitations

Several limitations must inform the interpretation of these findings. First, the absence of a randomised control group is the primary methodological limitation. Improvement on the post-test may partly reflect testing effects (familiarity with the instrument), Hawthorne effects (heightened engagement from study participation), or maturation — and these cannot be fully disaggregated from programme effects. This limitation was accepted because: withholding a potentially beneficial intervention from a group in which 100% of hypertensive participants were unaware of their condition would have been ethically unjustifiable; and because purposive sampling designs with same-day assessment are consistent with established standards for proof-of-concept evaluation of novel health promotion programmes in resource-limited settings [18].

Second, and most importantly, the same-day pre-test/post-test design captures maximum-engagement, immediate knowledge and attitude states but provides no evidence about the retention of knowledge gains or the durability of attitudinal and motivational changes beyond the programme day. Whether these proximal improvements translate into sustained behaviour change — dietary modification, increased physical activity — and ultimately into clinical risk reduction (blood pressure, BMI, lipid profiles) at 3, 6, or 12 months is unknown. This is the most significant gap in the current evidence base, and a structured 3–6-month follow-up study is the single highest priority for the next phase of HeartSmart research. The current findings should therefore be interpreted as evidence of programme efficacy at the immediate post-programme time-point, not as evidence of sustained behavioural change.

Third, the purposively selected, high-risk sample of *n* = 50 enhances internal validity for understanding programme effects in those most in need but substantially limits generalisability. Fourth, self-report measures for ABCD outcomes introduce social desirability bias risk, particularly on the post-test administered immediately after an intensive educational day. Fifth, single-site delivery at UNIJOS does not test programme effectiveness in the NSUK context or other Nigerian university settings.

### Implications for practice and policy

Despite these limitations, the HeartSmart Programme provides a validated, replicable model for campus-based CVD prevention appropriate for resource-limited settings across sub-Saharan Africa. The companion clinical assessment [9] and the present intervention study together constitute an evidence base for Nigerian university health services to implement systematic BP screening and structured health promotion within existing infrastructure. The high programme fidelity and peer educator model demonstrate that effective delivery does not require specialist staff, making scale-up within the university system feasible. The National Universities Commission and Federal Ministry of Health may draw on these findings in developing guidelines for integrated NCD prevention within university health services.

## Conclusions

The HeartSmart Programme demonstrates that a brief, one-day, theory-based, culturally tailored cardiovascular health promotion programme can achieve large improvements in CVD knowledge and meaningful attitudinal and motivational changes in high-risk students at a North-Central Nigerian university. The knowledge effect (d = 1.42) substantially exceeds international benchmarks; effects across all four ABCD domains were statistically significThis research received no specific external funding. The study was conducted as part of a doctoral dissertation at Nasarawa State University, Keffi, Nigeria. Clinical assessment equipment and programme materials were resourced by the principal investigaThis research received no specific external funding. The study was conducted as part of a doctoral dissertation at Nasarawa State University, Keffi, Nigeria. Clinical assessment equipment and programme materials were resourced by the principal investigaant. Findings are limited to immediate post-programme effects; whether these translate to sustained behaviour change and clinical risk reduction over time remains the most important unanswered question and the primary priority for future research.

Future research should prioritise: (a) randomised controlled trials to establish causal efficacy; (b) follow-up assessments at 3–6 months to evaluate knowledge retention and behavioural change; (c) scale-up evaluation at NSUK and other Nigerian universities; and (d) integration of biological outcome measures into pre-post designs. The HeartSmart Programme offers a practical, low-resource, culturally grounded blueprint for primordial CVD prevention in sub-Saharan African university settings.

## Supplementary Information

Supplementary Material 1.

## Data Availability

The datasets generated and analysed during the current study are available from the corresponding author on reasonable request.
