# Supplementary material for: HeartSmart: development and evaluation of a culturally tailored, theory-based cardiovascular health promotion programme for university students in a North-Central Nigerian university — a quasi-experimental pre-post study
Source: BMC Public Health. 2026 May 28;26:2275. doi: 10.1186/s12889-026-27944-7 (PMC13439998; doi:10.1186/s12889-026-27944-7)
Supplement: Supplementary file 1 [file 12889_2026_27944_MOESM1_ESM.docx]

# Appendix A: Study Questionnaire

The following instrument was used for data collection in both Phase 1 (cross-sectional survey, N=1,300) and Phase 2 (pre-test/post-test intervention evaluation, n=50). Sections B, C, and D (items 1–26) comprise the ABCD Risk Perception Questionnaire (Woringer et al., 2017; BMJ Open 7:e014413), adapted for use in the Nigerian university context following expert panel confirmation of item appropriateness without textual modification. Section A provides demographic and health behaviour data. Section E (Smoking cessation intentions) was included in the Phase 1 survey for comprehensive risk behaviour assessment.

**Section A: Respondent Characteristics**

Instruction: Circle the response/response number that corresponds to your answers or use the spaces provided.

1. Faculty ………………… 2. Department ………………………… Level ………

4. Religion: (1) Christian (2) Islam (3) Traditional (4) Other (Specify) ___________________

5. Age in years (at last birthday) …………………………………………

6. Gender: (1) Male (2) Female (3) Choose not to say

7. Marital status: (1) Single (2) Married (3) Choose not to say (4) Others ……………

8. Smoking Status: (1) I do not smoke (2) I have smoked before (3) I currently smoke

9. Alcohol drinking status: (1) I do not drink (2) I have drunk before (3) I currently drink

10. Have you ever been told by a health professional that you have high blood pressure? (1) Yes (2) No

11. Have you ever been told by a health professional that you have diabetes? (1) Yes (2) No

12. Do you have any immediate family members (parents or siblings) who have been diagnosed with or experienced a heart attack (myocardial infarction) or stroke? (1) Yes (2) No

13. In general, how would you rate your health status? (1) Very poor (2) Poor (3) Fair (4) Good (5) Excellent

**Section B: Knowledge on Cardiovascular Disease (ABCD Subscale A — Items 1–8)**

Instruction: Please indicate your response by ticking (√) in the appropriate box.

| **No.** | **Statement** | **True** | **False** | **Don't Know** |
| --- | --- | --- | --- | --- |
| 1 | One of the main causes of heart attack and stroke is stress |  |  |  |
| 2 | Walking and gardening are considered types of exercise that can lower the risk of having a heart attack or stroke |  |  |  |
| 3 | Moderately intense activity of 2.5 hours a week will reduce your chances of having a heart attack or stroke |  |  |  |
| 4 | People who have diabetes are at higher risk of heart attack or stroke |  |  |  |
| 5 | Managing your stress levels will help you to manage your blood pressure |  |  |  |
| 6 | Drinking high levels of alcohol can increase your cholesterol and triglyceride levels |  |  |  |
| 7 | HDL refers to 'good' cholesterol, and LDL refers to 'bad' cholesterol |  |  |  |
| 8 | A family history of heart disease is not a risk factor for high blood pressure |  |  |  |

**Section C: Perceived Risk of Heart Attack or Stroke (ABCD Subscale B — Items 9–16)**

Instruction: Please indicate your response by ticking (√) in the appropriate box.

| **No.** | **Statement** | **Strongly Agree** | **Agree** | **Disagree** | **Strongly Disagree** |
| --- | --- | --- | --- | --- | --- |
| 9 | I feel I will suffer from a heart attack or stroke some time during my life |  |  |  |  |
| 10 | It is likely that I will suffer from a heart attack or stroke in the future |  |  |  |  |
| 11 | It is likely that I will have a heart attack or stroke some time during my life |  |  |  |  |
| 12 | There is a good chance I will experience a heart attack or stroke in the next 10 years |  |  |  |  |
| 13 | My chances of suffering from a heart attack or stroke in the next 10 years are great |  |  |  |  |
| 14 | It is likely I will have a heart attack or stroke because of my past and/or present behaviours |  |  |  |  |
| 15 | I am not worried that I might have a heart attack or stroke |  |  |  |  |
| 16 | I am concerned about the likelihood of having a heart attack or stroke in the near future |  |  |  |  |

**Section D: Perceived Benefits and Intentions to Change (ABCD Subscale C — Items 17–23)**

Instruction: Please indicate your response by ticking (√) in the appropriate box.

| **No.** | **Statement** | **Strongly Agree** | **Agree** | **Disagree** | **Strongly Disagree** |
| --- | --- | --- | --- | --- | --- |
| 17 | I am thinking about exercising at least 2.5 hours a week |  |  |  |  |
| 18 | I intend or want to exercise at least 2.5 hours a week |  |  |  |  |
| 19 | When I exercise for at least 2.5 hours a week, I am doing something good for the health of my heart |  |  |  |  |
| 20 | I am confident that I can maintain a healthy weight by exercising at least 2.5 hours a week |  |  |  |  |
| 21 | I am not thinking about exercising for 2.5 hours a week |  |  |  |  |
| 22 | When I eat five portions of fruits and vegetables a day, I am doing something good for the health of my heart |  |  |  |  |
| 23 | Increasing my exercise to at least 2.5 hours a week will decrease my chances of having a heart attack or stroke |  |  |  |  |

**Section D: Healthy Eating Intentions (ABCD Subscale D — Items 24–26)**

Instruction: Please indicate your response by ticking (√) in the appropriate box.

| **No.** | **Statement** | **Strongly Agree** | **Agree** | **Disagree** | **Strongly Disagree** |
| --- | --- | --- | --- | --- | --- |
| 24 | I am confident that I can eat at least five portions of fruits and vegetables a day within the next 2 months |  |  |  |  |
| 25 | I am thinking about eating at least five portions of fruits and vegetables a day |  |  |  |  |
| 26 | I am not thinking about eating at least five portions of fruits and vegetables a day |  |  |  |  |

**Section E: Smoking Cessation Intentions (Phase 1 Survey Only — Items 27–31)**

Instruction: Please indicate your response by ticking (√) in the appropriate box. Pick N/A if you have NEVER smoked.

| **No.** | **Statement** | **Strongly Agree** | **Agree** | **Disagree** | **Strongly Disagree** | **N/A** |
| --- | --- | --- | --- | --- | --- | --- |
| 27 | I am thinking of stopping smoking within 2 months |  |  |  |  |  |
| 28 | I have reduced or stopped smoking |  |  |  |  |  |
| 29 | I intend or want to stop smoking |  |  |  |  |  |
| 30 | If I stop smoking, it will reduce my chances of having a heart attack or stroke |  |  |  |  |  |
| 31 | I am not thinking about stopping smoking |  |  |  |  |  |

Note. Items 1–26 (Sections B–D) comprise the ABCD Risk Perception Questionnaire (Woringer et al., 2017; BMJ Open). Items 27–31 (Section E) were included in the Phase 1 survey for comprehensive risk behaviour assessment; Section E was not included in the Phase 2 (HeartSmart intervention) pre-test/post-test evaluation as the study population had extremely low smoking prevalence (2.2% current smokers).
